# The "Cure" of Consumption

**Published:** 1901-07-13

**Authors:** 


### THE "CURE" OF CONSUMPTION.

On every hand we hear people speaking of the "cure" of consumption, and speaking of it, too, in terms which make it clear that they really imagine that a cure or restoration to health does actually take place in this disease. A proper understanding on this matter is of considerable importance, because in the reports issued by various sanatoria a strong tendency is visible to use the word "cure" in a manner which we cannot but think is unjustifiable, more especially as these reports are read, and indeed are intended to be read, by the lay public. As Dr. Theodore Williams recently remarked, in a lecture delivered at the London Polyclinic, when we read without explanation in these statistics that at one German sanatorium 11 per cent. of the patients were "cured" and 15·6 per cent. nearly cured, at another 22·23 were "apparently cured," and at another 81 per cent. of first-stage cases were "apparently cured," we hold our breath and wish to know a little more about these cases. Some sanatoria appear to be a little more modest, but one sanatorium, he says, "claims 81 per cent. 'apparently cured' and 100 per cent. 'improved'!"

If one thinks a moment of the pathological anatomy of the affair, and considers what is going on within the lung of a patient suffering from consumption, one looks with a certain degree of impatience upon a great deal which is constantly intruded upon us as to the "cure" of phthisis an impatience which one cannot but extend to those who allow themselves to use the English language in such a very Pickwickian sense. Let us speak rather of phthisis being arrested, and consider for a moment what we mean even by that expression. Dr. Williams, in the lecture above mentioned, tells us that in many instances grey or miliary tubercle is transformed into fibroid tissue so completely that it is difficult to imagine that this tissue can break down again. This, then, is a true arrest so far as the local tuberculous process is concerned. A certain mischief has been done, but the disease has been eliminated and nothing but a scar is left. But in many cases where a tuberculous mass has become quiescent and has become encapsuled in a fibrous envelope, such a mass is caseous and contains tubercle bacilli, and in such cases, although the disease may be spoken of as "arrested," it must be remembered that the virus is still there, and that these caseous masses may break down under various influences and lead to fresh local infection.

Another form of arrest is where a tuberculous nodule or mass becomes obsolescent, and is surrounded by chronic local pulmonary emphysema, which often prevents the detection of the tubercle by physical signs. "This is one of the commonest endings of tubercle, and the owner of the lungs which have gone through this process is generally a short-breathed individual, with a large, motionless chest, and often liable to wheezing. He is credited with being an asthmatic." Such patients may become fat and ruddy and present all the appearance of good health, barring the short breath. "Many eventually perish by an attack of hæmoptysis through the cracking of some degenerated vessel exposed in the old cavity, or the bursting of a small pulmonary aneurysm."

A not infrequent form of arrest is that in which a cavity situated in the upper lobe of one lung undergoes contraction. The increase of fibrosis causes a shrinking of the whole lung, and the walls of the cavity approximate and in some cases meet and cicatrise. Post-mortem observations show, however, that complete cicatrisation of a cavity is a very rare event. We often come across such cavities, says Dr. Williams, shrunk in capacity and with the bronchus leading to them blocked. Such a cavity would give rise to no auscultatory signs, and would yield no sputum to for analysis.

Then there is calcareous degeneration, which is often seen in necropsies, and specimens of which are often expectorated during life. This form of degeneration undoubtedly indicates the obsolescence of tubercle. But the expectoration of these calcareous masses must not be taken as proof of the arrest of tuberculosis, for at the same time as a patient may be expectorating cretaceous matter active disease may be progressing, and may indeed be the cause of the dislodgment of the chalky masses.

How far, then, may we properly consider these cases of arrested phthisis as cured, or even as "practically" cured? There are a number of young men and women, says Dr. Williams, who give a history of phthisical symptoms of some weeks or months, have bacilli in their sputum, and present physical signs of consolidation at one or both apices. Under appropriate treatment for half a year or a year they lose their symptoms, the tubercle bacilli in the sputum disappear together with most of their physical signs, and after high altitude treatment frequently all the signs. These patients are often classed as "cures." As for the pathological condition it is probable that the tubercle has undergone in part caseation, and in part fibrosis, and that their apices contain some small caseous masses embedded in fibrotic and healthy lung. No doubt in most of these patients the disease is arrested, but it cannot be called cured, for a change of surroundings from healthy to unhealthy, or overwork, or an attack of some lowering disease like influenza, will cause a return of the phthisical symptoms, tubercle bacilli will reappear in the sputum, softening of the apex will follow, and a cavity will be duly detected by physical signs, and this even after years of immunity. So much for arrest as shown by a restoration of general health as well as by physical signs; but it is to be noted that a mere improvement of general condition is but little to go by so long as signs remain showing that the local disease is active. "Though a patient may add one or even three stone to weight, may become as brown as a berry, and even regain strength, if the cough continues and signs of active mischief are still detected in the lungs, this general improvement may at any time vanish when the tubercle spreads or assumes fresh activity." This is a matter of much importance in interpreting the results of sanatorium treatment, for general improvement is far more common than local, and it is not always stated which is meant. Even the term arrest, then, "should only be applied when all general symptoms have ceased, when there is no longer cough and expectoration, night sweats or wasting, and the physical signs are either absent altogether, or, in the case of former cavities, signs of consolidation or contracted cavity have replaced those of the active cavity." During the next few weeks we shall no doubt hear a great deal about the cure of consumption. Let us not forget what sort of cure is possible and to what sort of arrest the word "cure" is only too often applied.

